# Impact of Temporary Storage Conditions on the Viability of Streptococcus pneumoniae in Saliva

**DOI:** 10.1128/msphere.00331-22

**Published:** 2022-11-21

**Authors:** Orchid M. Allicock, Anna York, Pari Waghela, Devyn Yolda-Carr, Daniel M. Weinberger, Anne L. Wyllie

**Affiliations:** a Department of Epidemiology of Microbial Diseases, Yale School of Public Health, New Haven, Connecticut, USA; CDC

**Keywords:** *Streptococcus pneumoniae*, carriage, pneumococcus, sample storage

## Abstract

Nasopharyngeal swabs are considered the gold-standard sample type for the detection of Streptococcus pneumoniae carriage, but recent studies have demonstrated the utility of saliva in improving the detection of carriage in adults. Saliva is generally collected in its raw, unsupplemented state, unlike nasopharyngeal swabs, which are collected into stabilizing transport media. Few data exist regarding the stability of pneumococci in unsupplemented saliva during transport and laboratory storage. We therefore evaluated the effect of storage conditions on the detection of pneumococci in saliva samples using strains representing eight pneumococcal serotypes. The bacteria were spiked into raw saliva from asymptomatic individuals, and we assessed sample viability after storage at 4°C, room temperature, and 30°C for up to 72 h; at 40°C for 24 h; and following three freeze-thaw cycles. We observed little decrease in pneumococcal detection following culture enrichment and quantitative PCR (qPCR) detection of the *piaB* and *lytA* genes compared to testing fresh samples, indicating the prolonged viability of pneumococci in neat saliva samples. This sample stability makes saliva a viable sample type for pneumococcal carriage studies conducted in remote or low-resource settings and provides insight into the effect of the storage of saliva samples in the laboratory.

**IMPORTANCE** For pneumococcal carriage studies, saliva is a sample type that can overcome some of the issues typically seen with nasopharyngeal and oropharyngeal swabs. Understanding the limitations of saliva as a sample type is important for maximizing its use. This study sought to better understand how different storage conditions and freeze-thaw cycles affect pneumococcal survival over time. These findings support the use of saliva as an alternative sample type for pneumococcal carriage studies, particularly in remote or low-resource settings with reduced access to health care facilities.

## INTRODUCTION

Streptococcus pneumoniae (pneumococcus) is a commensal organism that resides asymptomatically in the upper respiratory tract of healthy individuals. However, under certain circumstances, the pneumococcus can become pathogenic and is the causative agent of infections such as otitis media, sinusitis, and pneumonia as well as invasive pneumococcal diseases (IPDs) such as meningitis and sepsis. Pneumococcal pneumonia is the leading cause of respiratory infection morbidity and mortality globally, and the incidence is highest in young children, older adults, and immunocompromised individuals (1). Additionally, the case fatality rate from lower respiratory infections is highest in low- and middle-income countries and lowest in high-income countries (2, 3). Following the introduction of pneumococcal conjugate vaccines (PCVs), both asymptomatic carriage and IPD caused by vaccine serotypes declined. However, there was a concomitant increase in the prevalence of nonvaccine serotypes, known as serotype replacement (4). Postvaccine studies have provided insights into the shift in the frequency of vaccine and nonvaccine serotypes in both carriage and disease (5–8). Continued large-scale surveillance is needed to monitor the changing serotype landscape in order to better inform the development of next-generation vaccines.

Nasopharyngeal swabs are considered the gold standard for the detection of the carriage of pneumococci. While this approach works well in young children, a growing body of literature supports that this approach lacks sensitivity for the detection of carriage in adults. Accordingly, the WHO updated recommendations in 2013, advising for the additional collection of oropharyngeal swabs in older adults (9), yet the majority of studies continue to rely solely on nasopharyngeal swabbing. Due to their invasive nature, nasopharyngeal swabs are generally not well tolerated, and sample collection requires a health care worker. This sample burden means that nasopharyngeal swabs are a less desirable sample type for surveillance studies of longitudinal carriage within specific populations, particularly individuals who have restricted access to health care workers, such as the elderly, the disabled, or individuals in low-resource settings. Therefore, alternative, less-invasive sample types, which can be collected without the need for health care workers, are important for the study of pneumococcal carriage in these populations.

The challenges of nasopharyngeal swabbing were exemplified early in the coronavirus disease 2019 (COVID-19) pandemic response. Supply chain issues and safety concerns impacted the practicality and feasibility of pneumococcal carriage studies through 2020 and 2021. Nonessential in-person visits with health care workers were restricted due to severe acute respiratory syndrome coronavirus 2 (SARS-CoV-2) transmission concerns. Supply chain disruptions reduced access to materials required for nasopharyngeal and oropharyngeal sampling, further hampering attempts to continue pneumococcal carriage studies. These complications provided the impetus for pneumococcal carriage studies to move away from swab-based sampling and utilize alternative sample types. Saliva has been shown to be a sensitive sample type for the detection of pneumococcal carriage in individuals of all ages (10–12). Saliva sampling is noninvasive, and samples can be collected quickly and easily in the home, without a health care worker. However, at-home sample collection brings about potential issues with short-term storage, collection, and transport of the samples to the laboratory. In order to reap the benefits of the use of saliva samples for studies of pneumococcal carriage, more data are needed about how variations in storage conditions and the time from sample collection to laboratory processing may impact the detection and viability of pneumococci in these samples.

Therefore, we evaluated the viability of pneumococci in saliva over extended periods (up to 72 h) when stored at 4°C, room temperature, 30°C, or 40°C. We also investigated the viability of pneumococci after a series of freeze-thaw cycles at −20°C and −80°C, with and without glycerol supplementation, to evaluate the best practices for the storage and processing of saliva samples in the laboratory. Understanding the stability of pneumococci in saliva may prove particularly useful for carriage studies being conducted in remote regions or resource-limited settings where quick transport or access to cold storage may not be readily available.

## RESULTS

The viability of pneumococci in a sample was inferred by the detection of pneumococcal DNA after sample culture enrichment. Overall, the threshold cycle (*C_T_*) values obtained for both gene targets *piaB* and *lytA* were concordant (see Tables S1 to S3 in the supplemental material). For clarity, only *piaB* results are presented and discussed.

10.1128/msphere.00331-22.1 TABLE S1 Viability of S. pneumoniae in spiked saliva samples over a 72-h time course. Download Table S1, XLSX file, 0.02 MB. Copyright © 2022 Allicock et al. 2022 Allicock et al. https://creativecommons.org/licenses/by/4.0/ This content is distributed under the terms of the Creative Commons Attribution 4.0 International license.

### Viability of pneumococci over time.

**FIG 1:**
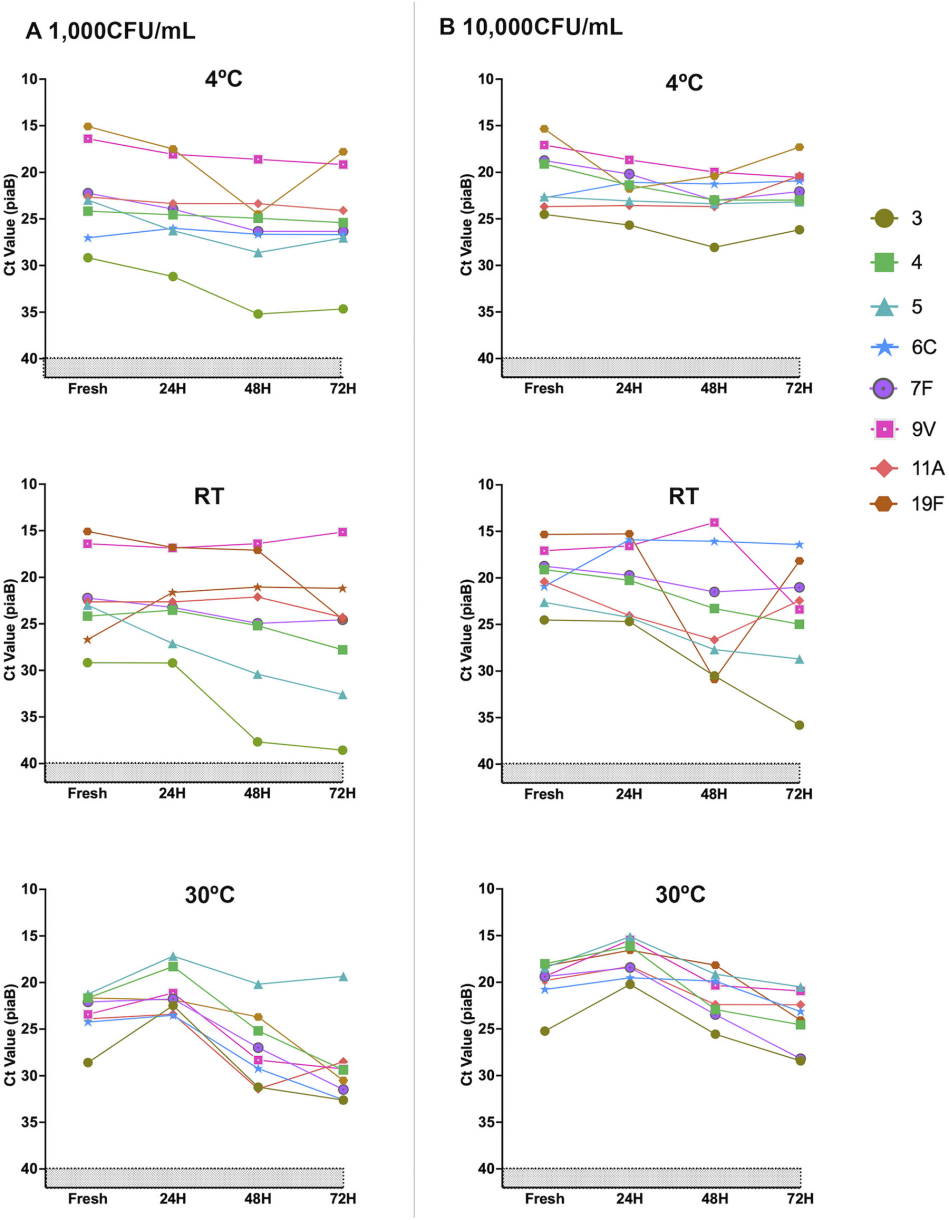
Viability of Streptococcus pneumoniae after prolonged storage at different temperatures. S. pneumoniae was detected in saliva spiked with 1,000 CFU/mL (A) and 10,000 CFU/mL (B) over a 72-h time course when stored at either 4°C, room temperature (RT), or 30°C. The detection of strains representing each serotype was relatively consistent for up to at least 48 h. Each point represents an individual sample. *lytA* values can be found in Table S1 in the supplemental material.

To examine the viability of pneumococci in raw saliva samples under temporary storage conditions, we measured the concentration of pneumococcal DNA following culture enrichment by quantitative PCR (qPCR). When comparing two concentrations of pneumococci in spiked saliva (10^3^ and 10^4^ CFU/mL), averaging across all serotypes at time zero, the *C_T_* values for spiked saliva at 10^3^ CFU/mL were on average 3.02-fold higher than those at 10^4^ CFU/mL (*P < *0.001) (Fig. 1). After adjusting for the concentration, there was no significant increase in the *C_T_* (which would represent a decline in bacterial survival) between 0 and 24 h (Δ*C_T_*, 0.05 [*P *= 0.94]); however, after 48 and 72 h, significant increases in the *C_T_* values were observed (Δ*C_T_* of 2.94 [*P < *0.001] and Δ*C_T_* of 3.78 [*P < *0.001], respectively). The pneumococci in saliva samples were slightly more stable at 4°C than at room temperature, but this was not significant (Δ*C_T_* of 0.17 [*P *= 0.76] and Δ*C_T_* of 0.10 [*P *= 0.87], respectively). Overall, despite some increases in *C_T_* values, pneumococci remained at detectable levels after 72 h for all serotypes at 4°C, room temperature, and 30°C, and at 40°C, only serotype 3 was not detected after 24 h (Table S1).

### Viability of pneumococci after freeze-thaw cycles.

**FIG 2:**
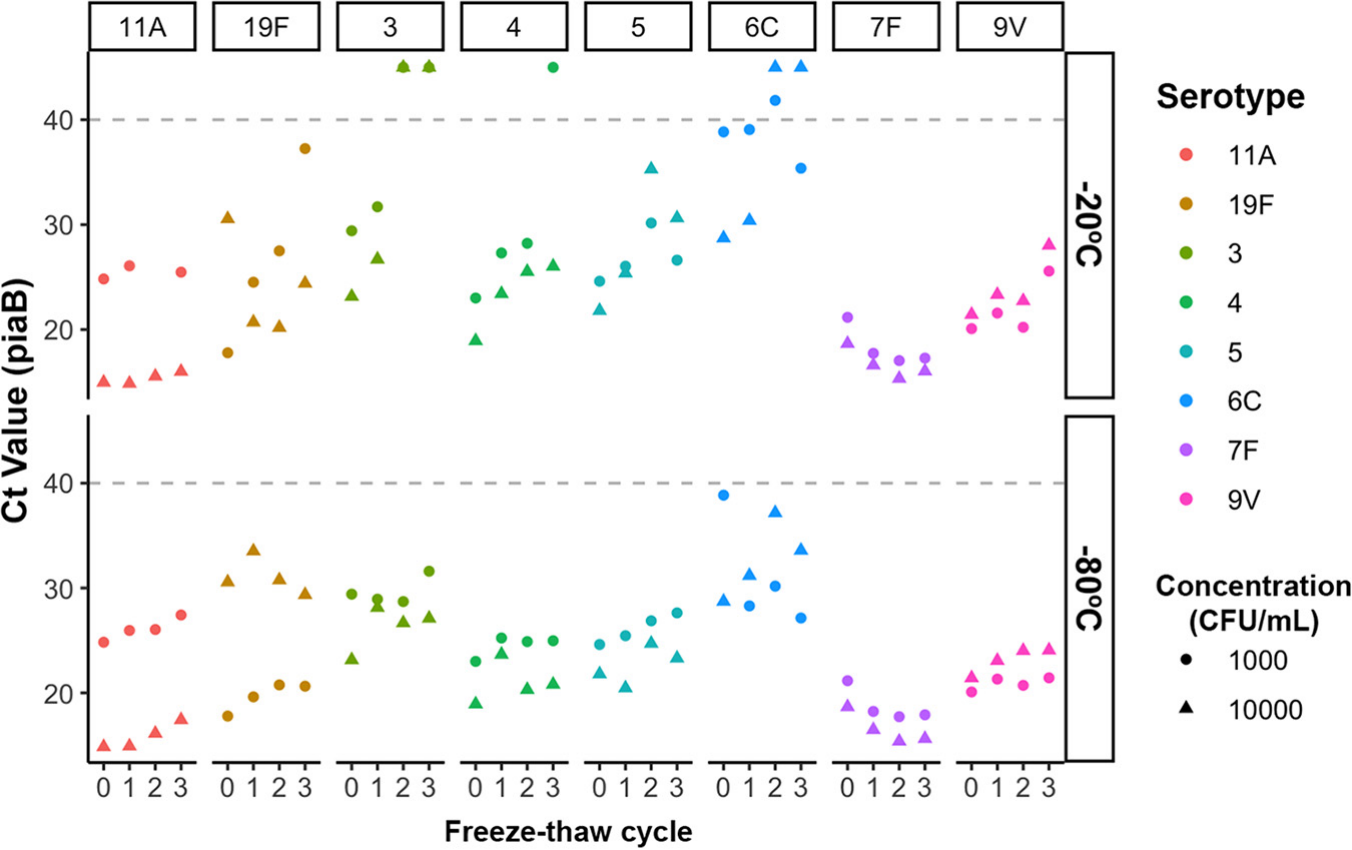
Viability of Streptococcus pneumoniae after freeze-thaw cycles. S. pneumoniae was detected in saliva spiked with 1,000 CFU/mL (circles) and 10,000 CFU/mL (triangles) over a series of three freeze-thaw cycles at either −20°C or −80°C. *lytA* values can be found in Table S2 in the supplemental material.

Under both conditions (−20°C and −80°C), there were no significant differences in *C_T_* values between fresh saliva and samples after the first freeze-thaw cycle for any of the eight serotypes tested (Fig. 2). However, pneumococcal loss (as inferred by increased *C_T_* values) was lower at −80°C than at −20°C for both cycle 2 (Δ*C_T_* of −3.81 at −80°C [*P *= 0.01] versus Δ*C_T_* of 3.877 at −20°C [*P < *0.001]) and cycle 3 (Δ*C_T_* of −4.65 at −80°C [*P < *0.05] versus Δ*C_T_* of 5.056 at −20°C [*P < *0.001]) compared to fresh saliva. The addition of glycerol as a cryoprotectant to the saliva samples before storage did not have an impact on survival at any stage, irrespective of the temperature (*P *= 0.1). No viable pneumococci were detected for serotypes 3 and 6C after cycle 2 or for serotype 4 after cycle 3 in the absence of glycerol, whereas only serotype 6C remained undetected in the presence of glycerol.

10.1128/msphere.00331-22.2 TABLE S2 Viability of S. pneumoniae after three freeze-thaw cycles. Download Table S2, XLSX file, 0.02 MB. Copyright © 2022 Allicock et al. 2022 Allicock et al. https://creativecommons.org/licenses/by/4.0/ This content is distributed under the terms of the Creative Commons Attribution 4.0 International license.

### Clinical validation.

**FIG 3:**
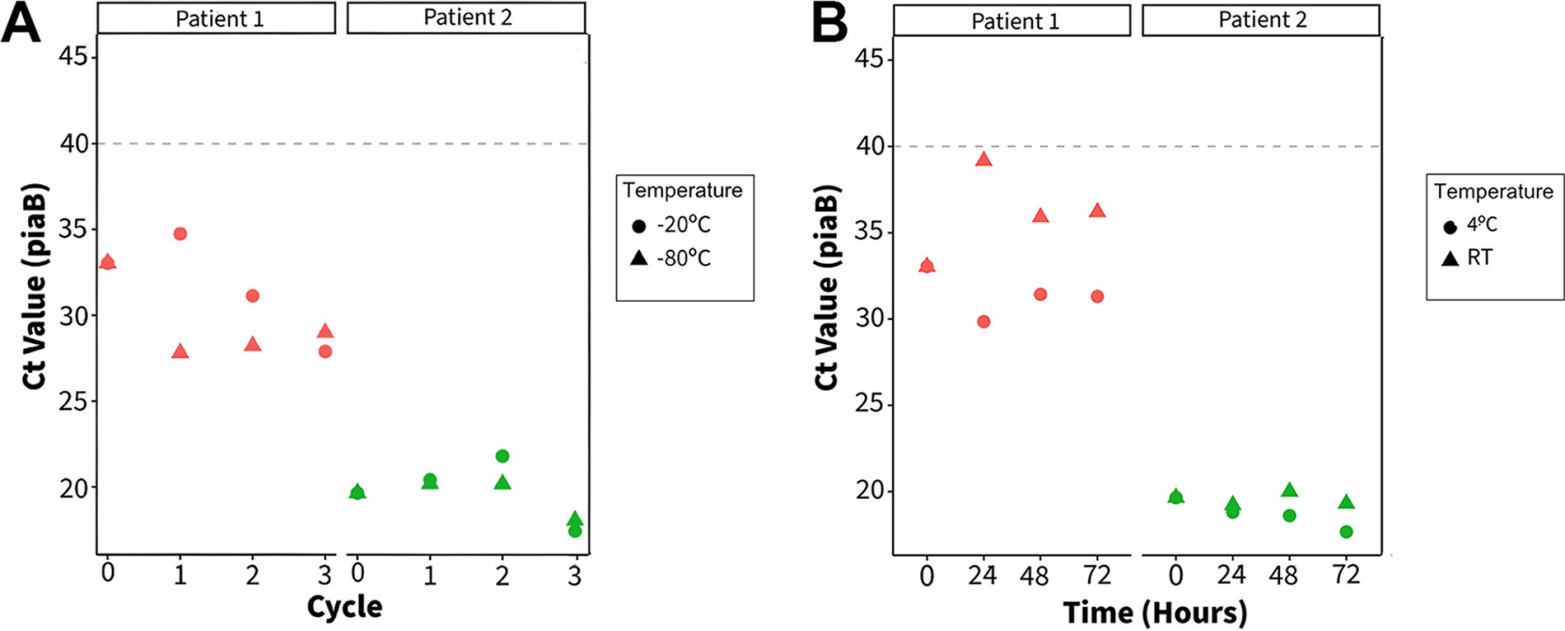
Detection of S. pneumoniae in clinical samples over three freeze-thaw cycles (A) and a 72-h time course (B). We were able to isolate multiple pneumococcal colonies from sample P2; however, we were unable to isolate colonies from P1. RT, room temperature. *lytA* values can be found in Table S3 in the supplemental material.

Pneumococci were detected in saliva specimens collected from two asymptomatic volunteers (referred to as P1 and P2). The *C_T_* value of sample P2 suggests that the pneumococcal concentration was between 10^3^ and 10^4^ CFU/mL, whereas P1 contained <10^1^ CFU/mL. The viability of pneumococci in both clinical samples remained stable over three freeze-thaw cycles and at both 4°C and room temperature over 72 h (Fig. 3). Notably, there was discordance between the *C_T_* values for the *piaB* and *lytA* genes (difference of >5 *C_T_* values) for sample P1, suggesting the possible carriage of nonpneumococcal streptococci with a *lytA* homologue (13–15) or potentially unencapsulated pneumococci (16) (Table S3).

10.1128/msphere.00331-22.3 TABLE S3 Viability of S. pneumoniae after extended storage of three pneumococcus-positive clinical saliva samples. Download Table S3, XLSX file, 0.01 MB. Copyright © 2022 Allicock et al. 2022 Allicock et al. https://creativecommons.org/licenses/by/4.0/ This content is distributed under the terms of the Creative Commons Attribution 4.0 International license.

An attempt was made to isolate pneumococci from both clinical samples. For sample P2, of 36 colonies that were optochin sensitive, 32 were identified to be the majority species belonging to serogroup 4, and 4/36 were identified to be a minority cocolonizing species belonging to serogroup 15. We were unable to isolate single colonies of pneumococci from sample P1 using standard culture-based methods.

## DISCUSSION

New PCVs that target different sets of pneumococcal serotypes are in various stages of clinical development and introduction in the community. Ongoing surveillance of pneumococci in the community is necessary to assess the impact of PCVs and monitor the emergence of nonvaccine serotypes. Nasopharyngeal swabs and/or oropharyngeal swabs are considered the gold-standard sample types for surveillance; however, they have their limitations. They are not well tolerated due to the discomfort of collection, resulting in many individuals showing reluctance or poor adherence to sampling protocols. Combined with the requirement for collection by trained health care workers, these challenges negatively impacted pneumococcal carriage surveillance studies during 2020 and 2021; an alternative approach was required. While saliva has been demonstrated to be a sensitive sample type for the detection of pneumococcal carriage (11, 12), the viability of pneumococci in raw, unsupplemented saliva had not been described.

For this study, we selected eight pneumococcal strains representing eight different serotypes (3, 4, 5, 6C, 7F, 9V, 11A, and 19F) with various degrees of encapsulation and different capsular properties. Serotypes 4, 11A, and 19F represent small, medium, and large capsules, respectively (17). Serotype 3 was included due to its clinical significance; it often causes severe disease and is able to evade the vaccine-induced immune response by releasing its capsule into the surrounding environment (18). While the majority of serotypes are anionic, serotype 7F (along with serotypes 7A, 14, 33F, 33A, and 37) is uncharged (19, 20). As we tested for the pneumococcal gene *piaB* to determine the presence and viability of pneumococci in saliva and since *piaB* is absent from the majority of unencapsulated pneumococci (16), we did not include any unencapsulated isolates in this study. Any future work assessing the viability of unencapsulated pneumococci may require alternate gene targets for their discrimination, such as SP2020 (15).

For the eight isolates tested, we demonstrated that saliva samples can be stored at room temperature or 4°C for up to 48 h (and often longer) without a major loss of viability of pneumococci. All strains of the eight serotypes tested showed no significant loss of survival over the first 24 h. Between 24 and 72 h, some decreases in survival were observed, representing a 10-fold loss of bacterial viability, but all strains were still detected regardless of the starting concentration. Therefore, for the storage of saliva collected for pneumococcal carriage studies, either room temperature or refrigeration is a feasible option during transportation.

In contrast, for some strains, multiple freeze-thaw cycles resulted in a decrease or, in some cases, a complete loss of detection, representing to us an effect on strain viability. Furthermore, we observed no benefit of the use of 10% glycerol as a cryoprotectant for this sample type. Taken together, these results suggest that for short-term storage (e.g., during transport to the laboratory), samples should not be frozen, and upon receipt at the laboratory, samples should be aliquoted for long-term freezer storage in order to minimize the number of freeze-thaw cycles that occur. Differences in the isolation of pneumococci from the two clinical samples (P1 and P2) were related to the concentrations of pneumococci in the saliva samples. Using culture-based methods, pneumococci could easily be isolated from sample P2 due to its higher concentration. Additional enrichment steps may be required for the isolation of pneumococci from sample P1 due to the reduced pneumococcal concentration and the high concentrations of other bacterial species in saliva (reflected in the high *C_T_* values) (21).

The key limitations of this study include the small sample size of clinical saliva samples tested and the method of spiking pneumococci into saliva. As we were collecting saliva from healthy adult donors, the number of samples positive for pneumococci was expected to be very low. The impact of storage conditions may also vary among host saliva samples, so our work could be extended to samples from a larger subject population with differing saliva microbial communities. The spiking protocol used meant that there were minute concentrations of brain heart infusion (BHI) medium with glycerol and phosphate-buffered saline (PBS) present in the spiked saliva samples. Nevertheless, the pneumococci in the saliva from the colonized individuals exhibited patterns of stability and viability similar to those of the spiked saliva samples.

The viability of pneumococci in raw, unsupplemented saliva under temporary storage conditions, as demonstrated here, further supports the use of saliva as a sample type for the detection of pneumococcal carriage. With few observed differences among the strains tested, these findings suggest that pneumococcal survival in saliva may be independent of capsule size and charge. However, since each serotype is represented by only one isolate, additional studies are recommended to further investigate this observation at the serotype level. The findings presented here are particularly important for studies conducted in low-resource settings and rural areas and for studies conducted in the absence of health care professionals and adequate cold-chain transfer of samples between sampling and processing sites. In addition to the ease of self-collection and less resource intensive, the use of saliva for pneumococcal carriage studies may increase participant enrollment and retention, which is particularly important for longitudinal studies.

## MATERIALS AND METHODS

### Ethics statement.

Deidentified saliva samples were collected from healthy adult volunteers who were asymptomatic for respiratory infection. Potential study participants were informed in writing about the purpose and procedure of the study and consented to study participation through the act of providing a saliva sample; the requirement for written informed consent was waived by the Institutional Review Board of the Yale Human Research Protection Program (Protocol ID 2000029374).

### Saliva samples.

Saliva samples were collected from healthy volunteers according to previously described protocols (12). Participants were asked to drool saliva into a 15-mL polypropylene tube at least 1 h after their last meal or drink; these saliva samples were considered whole-mouth unstimulated saliva. Samples were transferred at room temperature to the laboratory for temporary storage at 4°C and stored within 12 h at −80°C.

### Sample processing.

Each saliva sample was first tested for the presence or absence of pneumococci. After vigorous vortexing, a 50-μL aliquot of each sample was heated for 10 min at 95°C and then tested by qPCR targeting the pneumococcus-specific genes *piaB* and *lytA* (as described in detail below). Saliva samples that were qPCR negative for *piaB* were considered pneumococcus negative and were used in the spiking experiments. Samples that were qPCR positive for *piaB* were considered pneumococcus positive and are referred to as “clinical samples” here. Pneumococcus-negative saliva samples were stored at −20°C until required for spiking experiments.

### Bacterial isolates.

Pneumococcal isolates from our collection (Table 1) were selected to represent eight different serotypes (3, 4, 5, 6C, 7F, 9V, 11A, and 19F). These isolates were plated onto tryptic soy agar II (TSA II) plus 5% (vol/vol) defibrinated sheep blood (blood plates) and incubated at 37°C with 5% CO_2_ overnight. Serotypes were confirmed using Immulex pneumococcus antisera according to the manufacturer’s instructions. The lawn was harvested into 1 mL brain heart infusion (BHI) medium using a cotton swab and used to inoculate 45 mL BHI medium. Cultures were grown at 37°C with 5% CO_2_ to an optical density at 620 nm (OD_620_) of ~0.6 arbitrary units (AU). Cultures were harvested by centrifugation at 4,000 × *g*, and pellets were resuspended in 5 to 10 mL BHI medium supplemented with 10% (vol/vol) glycerol and stored at −80°C. The CFU per milliliter of each sample were determined by colony counting of serially diluted samples cultured on blood plates and incubated at 37°C with 5% CO_2_ overnight.

**TABLE 1:** Strains of Streptococcus pneumoniae used in this study

Strain	Serotype	Source or reference
ABC020026160	3	CDC
ABC020012001	4	CDC
ABC020024416	5	CDC
ABC020025076	6C	CDC
ABC010005954	7F	CDC
ABC010001831	9V	CDC
ABC020009080	11A	CDC
#64Israel	19F	26

### Experimental design.

For each isolate listed in Table 1, concentrated stocks were diluted in phosphate-buffered saline (PBS) to a suitable concentration and spiked into saliva at final concentrations of 10^3^ and 10^4^ CFU/mL, which are clinically relevant for saliva samples in children (10, 21). To determine the stability of pneumococci over time, clinical saliva samples were incubated at 4°C and room temperature (~20°C) only, while spiked samples were incubated at 4°C, room temperature, 30°C, or 40°C. At 24 h, 48 h, and 72 h, the saliva samples were vortexed, and 100 μL was removed from each sample for culture enrichment. Samples incubated at 40°C were tested only at the 24-h time point.

To determine the effect of freeze-thawing on the stability of pneumococci, saliva was spiked with pneumococci to final concentrations of 10^3^ and 10^4^ CFU/mL in both the presence and absence of glycerol added to a final concentration of 10% (vol/vol). Aliquots were stored for a minimum of 2 h at −20°C or −80°C before being thawed to room temperature. Once each of the aliquots was thawed, the samples were vortexed, and 100 μL was removed from each sample for culture enrichment before the remainder was returned to −20°C or −80°C. This process was repeated twice more. The unspiked, pneumococcus-negative donor saliva samples were also processed by this method as negative controls to further confirm the absence of pneumococci.

### Culture enrichment of saliva.

At each of the study time points, 100 μL of spiked saliva or clinical saliva was plated onto TSA II plus 5% (vol/vol) defibrinated sheep blood supplemented with 10 μg/mL gentamicin (Gent plates) (11) and incubated at 37°C with 5% CO_2_ overnight. The following day, all bacterial growth was harvested from culture plates into BHI medium plus 10% glycerol and stored at −80°C until further analysis. These stored samples were considered to be culture enriched for pneumococci. Culture enrichment of saliva samples at each time point ensures that the *C_T_* values obtained represent viable pneumococci and not exogenous DNA (10).

### Detection of pneumococcal carriage using the molecular method.

Culture-enriched saliva samples were thawed on ice. DNA was extracted from 200 μL of each sample using the MagMAX Ultra viral/pathogen nucleic acid isolation kit (Thermo Fisher Scientific) on the KingFisher Apex instrument (Thermo Fisher Scientific), with modifications (22). Each DNA template was tested by qPCR for the pneumococcal genes *piaB* (23, 24) and *lytA* (25). The assays were carried out in 20-μL reaction mixtures using SsoAdvanced universal probe supermix (Bio-Rad, USA); primer/probe mixes (Iowa black quenchers) for *piaB* and *lytA* at final concentrations of 10 nM and 12 nM, respectively; and 2.5 μL of genomic DNA. DNA of the S. pneumoniae serotype 19F strain was included in every run as a positive control. Assays were run on a CFX96 Touch instrument (Bio-Rad) under the following conditions: 95°C for 3 min followed by 45 cycles of 98°C for 15 s and 60°C for 30 s. Samples were considered positive for pneumococci when the *C_T_* values for both genes were ≤40 (10).

### Colony isolation and serotyping of pneumococcus-positive clinical samples.

Clinical samples that were qPCR positive for *piaB* (pneumococcus positive) were serially diluted in phosphate-buffered saline, and 100 μL of 10^−4^, 10^−5^, and 10^−6^ dilutions were then plated onto blood plates and incubated at 37°C with 5% CO_2_. Following incubation overnight, plates were screened for single alpha-hemolytic colonies, which were selected, expanded onto new plates, and tested for optochin sensitivity. Optochin-sensitive isolates were serotyped using Immulex pneumococcus antisera (SSI Diagnostica, Hillerød, Denmark) according to the manufacturer’s instructions.

### Statistical analysis.

To evaluate the impacts of the temperature, serotype, number of freeze-thaw cycles, and concentration of spiked bacteria on the recovery of pneumococci from spiked saliva samples, linear regression was used. Interaction terms were used to evaluate whether the effects of time, temperature, or starting concentration varied by strain. The Δ*C_T_* value represents the change in the *C_T_* value from freshly spiked saliva under each condition (categorical). *P* values of less than 0.05 were considered significant. Statistical tests were conducted using R (version 3.5.2) as described in the figure legends.
